# Oral human cytomegalovirus prevalence and its relationships with periodontitis and *Porphyromonas gingivalis* in Japanese adults: a cross-sectional study

**DOI:** 10.1590/1678-7757-2020-00501

**Published:** 2020-12-18

**Authors:** Mariko NAKAMURA, Hideo SHIGEISHI, SU Cheng-Yih, Masaru SUGIYAMA, Kouji OHTA

**Affiliations:** 1 Hiroshima University Graduate School of Biomedical and Health Sciences Program of Oral Health Sciences Hiroshima Japan Hiroshima University, Graduate School of Biomedical and Health Sciences, Program of Oral Health Sciences, Department of Public Oral Health, Hiroshima, Japan.; 2 Hiroshima University Graduate School of Biomedical and Health Sciences Program of Oral Health Sciences Hiroshima Japan Hiroshima University, Graduate School of Biomedical and Health Sciences, Program of Oral Health Sciences, Department of Oral Health Management, Hiroshima, Japan.

**Keywords:** Human Cytomegalovirus, Periodontitis, Porphyromonas gingivalis, Real-time PCR

## Abstract

**Objective:**

This study aimed to clarify the association between oral human cytomegalovirus (HCMV) and periodontitis in Japanese adults.

**Methodology:**

In total, 190 patients (75 men and 115 women; mean age, 70.2 years) who visited Hiroshima University Hospital between March 2018 and May 2020 were included. Oral rinse samples were taken to examine the presence of HCMV DNA using real-time polymerase chain reaction (PCR). *P. gingivalis* was detected by semi-quantitative PCR analysis.

**Results:**

HCMV DNA was present in nine of 190 patients (4.7%). There were significant associations between HCMV presence and the presence of ≥4-mm-deep periodontal pockets with bleeding on probing (BOP) (P<0.01) and ≥6-mm-deep periodontal pockets with BOP (P=0.01). However, no significant relationship was observed between HCMV presence and periodontal epithelial surface area scores. Logistic regression analysis revealed that the presence of ≥4-mm-deep periodontal pockets with BOP was significantly associated with HCMV (odds ratio, 14.4; P=0.01). Propensity score matching was performed between patients presenting ≥4-mm-deep periodontal pockets with BOP (i.e., active periodontitis) and patients without ≥4-mm-deep periodontal pockets with BOP; 62 matched pairs were generated. Patients who had ≥4-mm-deep periodontal pockets with BOP showed a higher rate of HCMV presence (9.7%) than those who lacked ≥4-mm-deep periodontal pockets with BOP (0.0%). There was a significant relationship between HCMV presence and ≥4-mm-deep periodontal pockets with BOP (P=0.03). A significant relationship was found between HCMV/*P. gingivalis* DNA presence and ≥4-mm-deep periodontal pockets with BOP (P=0.03).

**Conclusions:**

Coinfection of oral HCMV and *P. gingivalis* was significantly associated with active periodontitis. Moreover, interactions between oral HCMV and *P. gingivalis* may be related to the severity of periodontal disease.

## Introduction

Human cytomegalovirus (HCMV) is a double-stranded DNA virus belonging to the *Herpesviridae* family, along with herpes simplex virus, Epstein-Barr virus (EBV) and varicella zoster virus.^1^ HCMV has a 235-kb DNA genome encoding more than 165 open reading frames.^2^ Most children become infected with HCMV during the first year of life.^3^ HCMV generally remains latent in host cells throughout the lifespan of infected individuals, but it can be reactivated in immunocompromised hosts.^4^ HCMV infection is commonly regarded as a life-threatening infection in patients infected with human immunodeficiency virus and patients who have undergone organ transplantation.^5^

The oral cavity is a common site for primary human herpesvirus infection. HCMV prevalence has varied in patients with gingivitis or chronic periodontitis.^6,7^ Periodontitis is a polymicrobial infectious disease affecting gingiva, tooth-supporting connective tissue, and alveolar bone. Periodontal disease destroys the crevicular epithelium, allowing HCMV to infect epithelial cells in the periodontal pocket. Therefore, oral HCMV prevalence is associated with inflammation in periodontal tissue. HCMV has been found in unstimulated whole saliva from people with periodontitis, indicating that saliva may play a vital role in the transmission of HCMV to others.^8^ To the best of our knowledge, little is known regarding the relationship of HCMV with inflamed periodontal pocket/periodontal inflamed surface area (PISA) in Japanese adults. PISA is a useful indicator for evaluating the total amount of inflamed periodontal tissue.^9^ In this cross-sectional study, we investigated oral HCMV prevalence and its relationship with inflamed periodontal pockets. Furthermore, we investigated the association of HCMV with periodontal disease-related bacteria (i.e., *P. gingivalis*). The *fimA* fimbria of *P. gingivalis* is a virulence factor for *P. gingivalis*.^10^ Type II and IV *fimA* are closely associated with periodontal disease.^10^ Therefore, we investigated the *fimA* genotype to clarify the association between the HCMV prevalence and *fimA* genotype. Furthermore, we performed propensity score matching to analyze the relationship of HCMV with active periodontitis.

## Methodology

In this study, 201 patients who visited the Department of Oral Health of Hiroshima University Hospital between March 2018 and May 2020 were recruited. The study design was approved by the Research Ethics Committee of Hiroshima University (approval no. E-1115), and all patients provided written informed consent form. We included ≥20-year-old adults who had consented to participate in this study. Immunocompromised patients [i.e., patients who received chemotherapy (n=11), those with severe immunodeficiency (n=0), and those with autoimmune disease, who received immunosuppressant therapies (n=0)] were excluded, considering that such patients are thought to be more susceptible to microbial infection in the oral cavity compared with healthy individuals. Therefore, 190 patients (75 men and 115 women; mean age, 70.2 years) were included in this study. The estimated sample size required for Mann–Whitneys U test and χ^2^ test analyses, as determined using the G*Power (version 3.1.9.4, Heinrich-Heine-Universität Düsseldorf, Düsseldorf, Germany), were 134 and 88 patients, respectively; these estimations used a 5% significance level and 80% statistical power. Effect size (i.e., 0.5 for Mann–Whitneys U and 0.3 for χ^2^ test) was used for sample size estimation, in accordance with the approach described by Cohen.^11^

### Oral investigation

Probing depth and bleeding on probing (BOP) were assessed at six sites (i.e., mesiobuccal, mesiolingual, buccal, lingual, distobuccal, and distolingual) for each individual tooth by an experienced dentist. Intra-rater reliability of pocket probing measurement by the dentist was examined. The calculated value of intraclass correlation coefficient was 0.83, suggesting a reproducible assessment of pocket depth. The presence of bleeding at one site was recorded as a BOP-positive result. The PISA and periodontal epithelial surface area (PESA) scores were calculated in accordance with previously described method.^9^ Plaque control scores were determined by experienced dental hygienists in accordance with the method described by O’Leary et al., using a plaque-disclosing agent to examine dental plaque accumulation.^12^ The number of remaining teeth and use of dentures were also recorded.

### Oral rinse sample collection and DNA extraction

Oral rinse samples were collected by asking the patients to rinse their mouths with saline for 15 sec. Samples were collected in sterile plastic tubes and immediately centrifuged. The supernatant was decanted and the pellets were stored at -80°C for subsequent DNA extraction. DNA was extracted using a PureLink™ Microbiome DNA Purification kit (Thermo Fisher Scientific, Waltham, MA, USA), in accordance with the manufacturer’s protocol.

### HCMV DNA detection by real-time PCR

**Figure 1:**
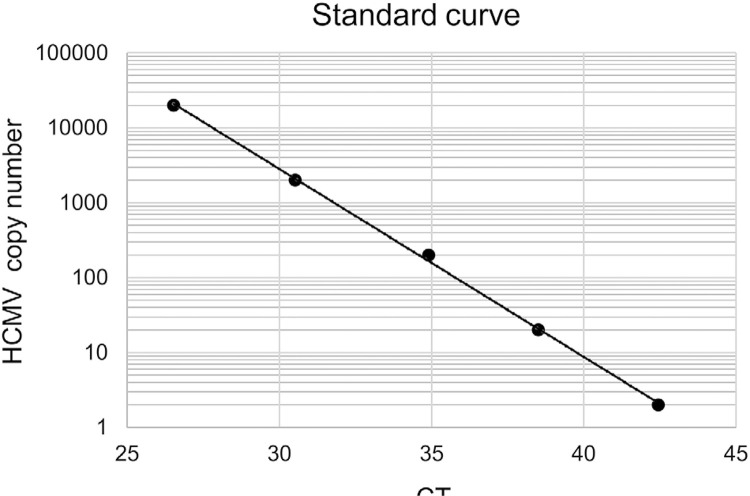
Standard curve indicating human cytomegalovirus (HCMV) copy number vs CT values

Real-time PCR analysis was performed to determine HCMV DNA copy number in samples using a CFX connect real-time PCR detection system (Bio-Rad, Hercules, CA, USA) in accordance with a previous study.^13^ The CMV PPS Set (Nippon Genetics Co., Ltd., Tokyo, Japan) was used, including PCR primer, probe, and standard PCR products, for HCMV DNA detection. HCMV strain AD169 was used to prepare the DNA control for PCR. Ten-fold dilutions of the standard PCR products (copy numbers ranging between 2.0 and 2.0×10^4^) were used to generate a standard curve of HCMV DNA, in accordance with the manufacturer’s protocol (Figure 1). Real-time PCR analysis was performed using FastGene™ QPCR Probe Mastermix w/ROX (Nippon Genetics Co., Ltd.). Amplification was performed with the following thermocycler protocol: 95°C for 10 min; followed by 50 cycles of 95°C for 10 sec and 62°C for 1 min; and then 40°C for 30 sec. Copy numbers above the detection limit in a standard curve for HCMV DNA were considered as positive results.

A standard curve was generated estimated by plotting obtained CT values against HCMV copy numbers ranging between 2.0 and 2.0x104.

### *P. gingivalis* detection and *fimA* genotyping by PCR

In accordance with the method used in our previous study, *P. gingivalis* was detected by PCR with specific DNA primer sets.^14^ Furthermore, the *fimA* genotype of *P. gingivalis* was examined using specific primers, as described previously.^15^ PCR products were amplified with GoTaq^®^ Green Master Mix (Promega, Madison, WI, USA). Amplification was performed with the following thermocycler protocol: initial melting at 95°C for 2 min; followed by 30 cycles of 95°C for 1 min, 57°C for 1 min, and 72°C for 1 min. PCR products were electrophoresed on 2% agarose gels and stained with ethidium bromide. The presence of PCR product in the expected size was regarded as a positive result.

### Statistical analysis

The Mann–Whitneys U test was used to compare differences in clinical parameters between groups. When necessary χ^2^ test or Fisher’s exact test were used. We performed residual analysis to determine which cell had a significant difference in the χ^2^ test for comparison between four groups. If the adjusted standardized residue was ≥1.96, the cells were considered to present significantly more subjects than expected. Bimodal logistic regression analysis was conducted by a step-wise model, using HCMV as the dependent variable; variables with a P-value inferior to 0.2 in univariate analysis were used as independent variables. The Hosmer–Lemeshow test result was not statistically significant (i.e., P>0.05), suggesting that the model exhibited good fitness. A propensity score-matched analysis was performed to adjust for confounders effects. Propensity scores were calculated by logistic regression analysis of 10 clinical factors (i.e., age, sex, hypertension, diabetes, hyperlipidemia, stroke, heart disease, smoking, remaining teeth, and denture use). Caliper of 0.25 standard deviations of the propensity score was used for analysis. Statistical analysis was performed using SPSS Statistics, version 24.0 (IBM Corp., Armonk, NY, USA). P<0.05 indicated a statistically significant difference.

## Results

### Association between HCMV DNA presence and clinical factors

HCMV DNA was present in nine of 190 patients (4.7%). Table 1 summarizes the relationships of HCMV DNA presence with clinical factors. Men presented significantly higher rate of HCMV DNA presence, compared with women (P=0.03, Fisher’s exact test). However, no significant relationships were found between HCMV DNA presence and other clinical factors (i.e., age, medical history, smoking, remaining teeth, and denture use).

**Table 1:** Associations of oral HCMV DNA with clinical parameters

Clinical factor (n)	HCMV DNA		P-value
	(-) (n=181)	(+) (n=9)	
**Age**	70.2±11.4	69.0±10.9	0.77
**Age in years**			0.63
30-39 (2)	2 (100%)	0 (0.0%)	
40-49 (7)	6 (85.7%)	1 (14.3%)	
50-59 (22)	22 (100%)	0 (0.0%)	
60-69 (51)	47 (92.2%)	4 (7.8%)	
70-79 (67)	65 (97.0%)	2 (3.0%)	
80-89 (38)	36 (94.7%)	2 (5.3%)	
90-99 (3)	3 (100%)	0 (0.0%)	
**Sex**			
Men (75)	68 (90.7%)	7 (9.3%)	0.03
Women (115)	113 (98.3%)	2 (1.7%)	
**Heart disease**			
No (179)	171 (95.5%)	8 (4.5%)	0.42
Yes (11)	10 (90.9%)	1 (9.1%)	
**Stroke Diabetes**			
No (184)	176 (95.7%)	8 (4.3%)	0.26
Yes (6)	5 (83.3%)	1 (16.7%)	
**Hypertension**			
No (142)	136 (95.8%)	6 (4.2%)	0.69
Yes (48)	45 (93.8%)	3 (6.3%)	
**Diabetes**			
No (166)	157 (94.6%)	9 (5.4%)	0.61
Yes (24)	24 (100%)	0 (0.0%)	
**Hyperlipidemia**			
No (155)	147 (94.8%)	8 (5.2%)	1.0
Yes (35)	34 (97.1%)	1 (2.9%)	
**Smoking**			
Non-smoker (161)	154 (95.7%)	7 (4.3%)	0.63
Current/former smoker (29)	27 (93.1%)	2 (6.9%)	
**Remaining teeth**	23.4±6.2	20.7±6.4	0.07
**Denture user**			
Non-user (142)	135 (95.1%)	7 (4.9%)	1.0
User (48)	46 (95.8%)	2 (4.2%)	

P values less than 0.05 were considered statistically significant.

Abbreviation: HCMV, human cytomegalovirus.

### Association of HCMV DNA presence with dental plaque accumulation and periodontal health condition

Following, associations of HCMV DNA presence with plaque control score and periodontal health condition were assessed (Table 2). Plaque control scores were higher in patients with HCMV DNA than in patients without HCMV DNA; however, this difference was not statistically significant. A total of 67 of 190 (35.3%) patients had ≥4-mm-deep periodontal pockets with BOP (i.e., active periodontitis). Six of the 65 (9.2%) patients with ≥6-mm-deep periodontal pockets presented HCMV DNA presence; no significant association was found between HCMV DNA presence and probing depth. Patients who had ≥4-mm-deep periodontal pockets with BOP demonstrated a higher rate of HCMV DNA presence (11.9%), compared with patients who lacked ≥4-mm-deep periodontal pockets with BOP (0.8%). Furthermore, patients who had ≥6-mm-deep periodontal pockets with BOP demonstrated a higher rate of HCMV DNA presence (13.3%) in comparison with patients without ≥6-mm-deep periodontal pockets with BOP (2.1%). There was a significant association of HCMV DNA presence with ≥4-mm-deep periodontal pockets with BOP (P<0.01, Fisher’s exact test), as well as with ≥6-mm periodontal pockets with BOP (P=0.01, Fisher’s exact test). The mean values of PISA and PESA were higher in patients without HCMV DNA than in patients with HCMV DNA; however, these differences were not statistically significant. *Post-hoc* statistical analysis revealed that the statistical power of the χ^2^ test was *≥*0.8 (i.e., high statistical power), but the statistical power of the Mann-Whitneys U-test was <0.8 (i.e., low statistical power). These results suggested a possible β error due to the low prevalence of HCMV.^11^ Therefore, it was necessary to consider the probability of a β error when assessing the results of PCR, PISA, and PESA statistical analyses.

**Table 2:** Associations of oral HCMV DNA with periodontal condition

Factor (n)	HCMV DNA		P-value
	(-)	(+)	
**Plaque Control Record scores (%)**	35.1±18.2	41.5±18.4	0.33
**Probing depth**			
<4 mm (75)	74 (98.7%)	1 (1.3%)	0.09
≥4 mm and <6 mm (50)	48 (96.0%)	2 (4.0%)	
≥6 mm (65)	59 (90.8%)	6 (9.2%)	
**≥4 mm periodontal pocket with BOP**			
No (123)	122 (99.2%)	1 (0.8%)	<0.01
Yes (67)	59 (88.1%)	8 (11.9%)	
**≥6 mm periodontal pocket with BOP**			
No (145)	142 (97.9%)	3 (2.1%)	0.01
Yes (45)	39 (86.7%)	6 (13.3%)	
**PISA (mm**^**2**^**)**	201.0±215.9	114.8±85.1	0.46
**PESA (mm^2^)**	1219.7±1003.1	1071.6±349.5	0.84

P values less than 0.05 were considered statistically significant. Abbreviations: HCMV, human cytomegalovirus; *P. gingivalis, Porphyromonas gingivalis*; PISA, periodontal inflamed surface area; PESA, periodontal epithelial surface area; BOP, bleeding on probing.

### Associations of oral HCMV/*P. gingivalis* with periodontal health condition

Associations of HCMV/*P. gingivalis* DNA presence with the plaque control score and periodontal health condition were assessed (Table 3). A significant relationship was found between HCMV/*P. gingivalis* DNA presence and probing depth or ≥4-mm/≥6-mm-deep periodontal pockets with BOP. Residual analysis revealed that HCMV-negative/*P. gingivalis*-positive and HCMV-positive/*P. gingivalis*-positive groups presented significantly more ≥4-mm-deep periodontal pockets with BOP. Additionally, the HCMV-positive/*P. gingivalis*-positive group presented significantly more ≥6-mm-deep periodontal pockets with BOP. These results suggest that the presence of both HCMV and *P. gingivalis* is associated with deep periodontal pockets with BOP.

**Table 3:** Associations of oral HCMV/*P. gingivalis* with periodontal condition

Factor (n)	HCMV(-)/*P. gingivalis*(-) (n=86)	HCMV(+)/ *P. gingivalis*(-) (n=1)	HCMV(-)/*P. gingivalis*(+) (n=95)	HCMV(+)/ *P. gingivalis*(+) (n=8)	P-value
**Plaque Control Record scores (%)**	32.1±19.7	25.0	37.6±16.7	44.2±18.5	0.10
**Probing depth**					
<4 mm (75)	46 (53.5%)*	1 (0.0%)	28 (29.5%)	0 (0.0%)	0.01
≥4 mm and <6 mm (50)	22 (25.6%)	0 (0.0%)	26 (27.4%)	2 (25.0%)	
≥6 mm (65)	18 (20.9%)	0 (0.0%)	41 (43.2%)*	6 (75.0%)*	
**≥4 mm periodontal pocket with BOP**					
No (123)	67 (77.9%)*	1 (100%)	55 (57.9%)	0 (0.0%)	<0.01
Yes (67)	19 (22.1%)	0 (0.0%)	40 (42.1%)*	8 (100%)*	
**≥6 mm periodontal pocket with BOP**					
No (145)	74 (86.0%)*	1 (100%)	68 (71.6%)	2 (25.0%)	0.01
Yes (45)	12 (14.0%)	0 (0.0%)	27 (28.4%)	6 (75.0%)*	
**PISA (mm^2^)**	173.8±148.1	180.8	227.3±264.2	101.6±88.0	0.78
**PESA (mm^2^)**	1127.1±304.5	1017	1306.0±1374.2	1080.8±382.0	0.98

P values less than 0.05 were considered statistically significant. *Adjusted standardized residue was greater than or equal to 1.96. Abbreviations: HCMV, human cytomegalovirus; *P. gingivalis, Porphyromonas gingivalis*; PISA, periodontal inflamed surface area; PESA, periodontal epithelial surface area; BOP, bleeding on probing.

### Association of HCMV DNA presence with *fimA* genotypes of *P. gingivalis*

Out of 190 patients, 103 (54.2%) had *P. gingivalis*. Associations of HCMV DNA presence with *fimA* genotypes are summarized in Table 4. Type II *fimA* was the most common genotype among six genotypes in patients with *P. gingivalis*. However, no significant relationships were found between HCMV DNA presence and *fimA* genotypes.

**Table 4:** Associations of oral HCMV DNA with *P. gingivalis* and *fimA* genotypes

Factor (n)	HCMV DNA		P-value
	(-)	(+)	
*P. gingivalis*			
Negative (87)	86 (98.9%)	1 (1.1%)	0.04
Positive (103)	95 (92.2%)	8 (7.8%)	
**Type I *fimA***			
Negative (85)	80 (94.1%)	5 (5.9%)	0.14
Positive (18)	15 (83.3%)	3 (16.7%)	
**Type Ib *fimA***			
Negative (90)	83 (92.2%)	7 (7.8%)	1.0
Positive (13)	12 (92.3%)	1 (7.7%)	
**Type II *fimA***			
Negative (2)	2 (100%)	0 (0.0%)	1.0
Positive (101)	93 (92.1%)	8 (7.9%)	
**Type III *fimA***			
Negative (98)	90 (91.8%)	8 (8.2%)	1.0
Positive (5)	5 (100%)	0 (0.0%)	
**Type IV *fimA***			
Negative (90)	82 (91.1%)	8 (8.9%)	0.59
Positive (13)	13 (100%)	0 (0.0%)	
**Type V *fimA***			
Negative (100)	92 (92.0%)	8 (8.0%)	1.0
Positive (3)	3 (100%)	0 (0.0%)	

P values less than 0.05 were considered statistically significant. Abbreviations: HCMV, human cytomegalovirus; *P. gingivalis, Porphyromonas gingivalis*; BOP, bleeding on probing.

### Independent clinical factors associated with HCMV by logistic regression analysis

Logistic regression analysis was performed to identify independent factors associated with HCMV DNA, using clinical parameters with P<0.2 (i.e., sex, remaining teeth, probing depth, ≥4-mm-deep periodontal pockets with BOP, and *P. gingivalis* presence) as independent variables. P values for the Hosmer–Lemeshow test were 0.23, indicating a good fit of the model. Logistic regression analysis demonstrated that the presence of ≥4-mm-deep periodontal pockets with BOP was significantly associated with HCMV presence (odds ratio, 14.4; 95% confidence interval, 1.74–119.68; P=0.01).

### Relationship of active periodontitis with HCMV DNA presence in propensity score-matched patients

Propensity score-matching was performed between patients who had ≥4-mm-deep periodontal pockets with BOP (i.e., patients with active periodontitis) and those who lacked ≥4-mm-deep periodontal pockets with BOP (i.e., patients without active periodontitis) using propensity scores generated based on 10 clinical factors: age, sex, heart disease, stroke, hypertension, diabetes, hyperlipidemia, smoking, remaining teeth, and denture use. In total, 124 propensity score-matched patients (62 patients in matched pairs) were assessed by univariate analysis. None of the 10 clinical variables were significantly associated with HCMV DNA presence (Table 5). Then, associations were investigated between deep periodontal pockets with bleeding and HCMV DNA or *P. gingivalis* presence. Patients who had ≥4-mm-deep periodontal pockets with BOP showed a higher rate of HCMV DNA presence (9.7%) in comparison with patients who lacked ≥4-mm-deep periodontal pockets with BOP (0.0%) (P=0.03, Fisher’s exact test) (Table 6). Also, a significant relationship was observed between *P. gingivalis* presence and ≥4-mm-deep periodontal pockets with BOP (P<0.01, Fisher’s exact test) (Table 6). Furthermore, a significant relationship was found between HCMV/*P. gingivalis* DNA presence and ≥4-mm-deep periodontal pockets with BOP (P=0.03).

**Table 5:** Patients’ characteristics according to the presence of deep periodontal pockets with bleeding; comprising 124 propensity score-matched patients.

Clinical factor (n)	Patients without ≥4 mm periodontal pocket exhibiting BOP (n=62)	Patients with ≥4 mm periodontal pocket exhibiting BOP (n=62)	P-value
**Age**	68.4±10.5	69.7±11.3	0.34
**Age in years**			
30-39 (2)	1 (50.0%)	1 (50.0%)	0.95
40-49 (5)	2 (40.0%)	3 (60.0%)	
50-59 (14)	8 (57.1%)	6 (42.9%)	
60-69 (37)	20 (54.1%)	17 (45.9%)	
70-79 (45)	22 (48.9%)	23 (51.1%)	
80-89 (21)	9 (42.9%)	12 (57.1%)	
**Sex**			
Male (57)	29 (50.9%)	28 (49.1%)	1.0
Female (67)	33 (49.3%)	34 (50.7%)	
**Heart disease**			
No (117)	58 (49.6%)	59 (50.4%)	1.0
Yes (7)	4 (57.1%)	3 (42.9%)	
**Stroke**			
No (120)	60 (50.0%)	60 (50.0%)	1.0
Yes (4)	2 (50.0%)	2 (50.0%)	
**Hypertension**			
No (90)	44 (48.9%)	46 (51.1%)	0.84
Yes (34)	18 (52.9%)	16 (47.1%)	
**Diabetes**			
No (108)	54 (50.0%)	54 (50.0%)	1.0
Yes (16)	8 (50.0%)	8 (50.0%)	
**Hyperlipidemia**			
No (99)	49 (49.5%)	50 (50.5%)	1.0
Yes (25)	13 (52.0%)	12 (48.0%)	
**Smoking**			
Non-smoker (99)	49 (49.5%)	50 (50.5%)	1.0
Current/former smoker (25)	13 (52.0%)	12 (48.0%)	
**Remaining teeth**	23.5±5.9	23.3±6.0	0.70
**Denture user**			
Non-user (93)	47 (50.5%)	46 (49.5%)	1.0
User (31)	15 (48.4%)	16 (51.6%)	

P values less than 0.05 were considered statistically significant. Abbreviation: BOP, bleeding on probing.

**Table 6:** Deep periodontal pockets with bleeding: associations with HCMV and *P. gingivalis* in 124 propensity score-matched patients

Clinical factor (n)	Patients without ≥4 mm periodontal pocket exhibiting BOP (n=62)	Patients with ≥4 mm periodontal pocket exhibiting BOP (n=62)	P-value
**HCMV**			
Negative (118)	62 (52.5%)	56 (47.5%)	0.03
Positive (6)	0 (0.0%)	6 (100%)	
***P. gingivalis***			
Negative (55)	37 (67.3%)	18 (32.7%)	<0.01
Positive (69)	25 (36.2%)	44 (63.8%)	
**HCMV(+)/*P. gingivalis*(+)**			
Yes (6)	0 (0.0%)	6 (100%)	0.03
No (118)	62 (52.5%)	56 (47.5%)	

P values less than 0.05 were considered statistically significant.Abbreviations: HCMV; human cytomegalovirus; *P. gingivalis, Porphyromonas gingivalis*; BOP, bleeding on probing.

## Discussion

In a recent review, Slots reported that oral HCMV infection occurred in 40% of patients with chronic periodontitis and 6% in patients with healthy periodontal tissue.^7^ However, a low percentage of HCMV-positive cases was found in this study. Notably, many participants presented a stable periodontal condition without periodontal inflammatory signs or localized periodontal inflammation. Furthermore, immunocompromised patients were excluded from this study. These factors may be related to the low prevalence of HCMV. A previous meta-analysis suggested that oral HCMV and periodontitis are significantly associated.^6^ Notably, logistic regression analysis demonstrated that deep periodontal pockets with bleeding constitutes a significant factor for oral HCMV prevalence. Furthermore, deep periodontal pockets with bleeding was significantly associated with the presence of HCMV DNA in propensity score-matched patients. Additionally, a significant association was found between the presence of both HCMV DNA and *P. gingivalis* and deep periodontal pockets with BOP. Our results strongly suggest that coinfection of oral HCMV and *P. gingivalis* is associated with active periodontitis in middle-aged and older Japanese people (mean age in this study: 70.2 years).

The association between PISA and oral HCMV has not been fully elucidated so far. In this study, no significant association was found between PISA and oral HCMV prevalence. Oral HCMV may be involved in local periodontal inflammation rather than total periodontal inflammation. Additionally, the low prevalence of HCMV may have caused false negative results in the statistical analysis of PISA. A further study will be required to clarify the association between oral CMV and PISA using a large number of HCMV-positive cases. Furthermore, the presence of HCMV in inflamed periodontal pockets remains unclear. Additional investigation using resected inflamed periodontal tissue or crevicular fluid is necessary to identify the existence of HCMV in periodontal pockets.

Regarding oral herpes viruses other than HCMV, EBV is presumed to establish lifelong latent infections in the oral cavity.^16^ The specific histological structure of the tonsillar region may facilitate EBV infection, for lymphoid tissue is abundant in the tonsillar region. EBV can infect B lymphocytes through saliva. It can also infect epithelial cells.^16^ EBV amplification occurs in epithelial cells, followed by shedding in saliva.^17^ Thus, the oral cavity may play a major role in virus transmission to the nasopharynx and gastrointestinal tract. HCMV has been identified in oral epithelial cells by histochemical analysis.^18^ Thus, HCMV possibly infect epithelial cells through ulcerated gingival epithelium of the periodontal pocket. Considering the significant associations reported between EBV/HCMV prevalence and periodontitis,^7,19,20^ inflamed periodontal pockets may serve as a main reservoir of these herpes viruses in the oral cavity.

Regarding the interaction between herpes viruses and bacteria related to periodontal disease, butyric acid produced by *P. gingivalis* has been shown to induce EBV reactivation via transcriptional activation of the *BZLF1* gene.^21^ Butyric acid blocks the enzymatic activity of histone deacetylases by competing with histone deacetylase substrate binding at the catalytic site of the enzyme.^22^ Furthermore, *P. gingivalis* has been shown to induce human immunodeficiency virus-1 reactivation by chromatin modification, indicating that periodontal anaerobic bacteria may contribute to activation of latent viruses.^23^ These results highlight the significance of periodontopathic bacteria as activator of viral organisms.

Proinflammatory cytokines and chemokines released by gingival fibroblasts infected with HCMV attract cytotoxic T cells and natural killer cells.^7^ In addition, pro-inflammatory cytokines may activate matrix metalloproteinases, resulting in periodontal ligament destruction and osteoclastic alveolar bone resorption.^7^ HCMV enhances immunosuppression by inhibiting cell surface expression of MHC class I molecules and activity of natural killer cells.^24^ Therefore, HCMV considerably affects periodontal tissue destruction by inducing local proinflammatory cytokines and impairing the local host immune response. In this study, the presence of oral HCMV and *P. gingivalis* were significantly associated. Coinfection of herpes virus and bacteria related to periodontal disease increases the risk of chronic periodontitis.^25,26^ Moreover, HCMV induces the adherence of *Actinobacillus actinomycetemcomitans* to periodontal epithelial cells, suggesting that HCMV can enhance the virulence of bacteria related to periodontal disease.^27^ Therefore, HCMV causes severe periodontitis by enhancing the pathogenicity of periodontal bacteria.

*P. gingivalis* produces several virulence factors (i.e., lipopolysaccharide, hemagglutinins, capsule, fimbriae, protease, gingipains, and others). Previous studies have demonstrated that lipopolysaccharide, fimbriae, and gingipains of *P. gingivalis* are significantly involved in periodontitis progression.^28^*P. gingivalis* has the potential to destroy periodontal tissues with lipopolysaccharide-induced inflammatory cytokines, providing the HCMV with the chance to infect periodontal tissues. Therefore, HCMV is thought to depend on coinfection with *P. gingivalis*. Envelope glycoprotein B is a potential virulence factor of HCMV.^29^ HCMV may also enhance the virulence of *P. gingivalis* by inhibiting the local host immune response.

This study had limitations. Clinical attachment loss can be used to evaluate the severity of periodontitis. However, the attachment level of the teeth was not measured in many participants. Therefore, the relationship between HCMV prevalence and the severity of periodontitis remains unclear. Furthermore, the small number of HCMV-positive cases may have affected the results of statistical analyses of plaque accumulation and the periodontal inflamed surface area.

In conclusion, oral HCMV was significantly associated with deep periodontal pockets with bleeding. We found that coinfection of oral HCMV and *P. gingivalis* is important for active periodontitis. Moreover, interactions between oral HCMV and *P. gingivalis* may be involved in the severity of periodontal disease. It is important to understand the relationship between oral herpes viruses and bacteria related to periodontal disease in the pathology of periodontitis. Further prospective studies are necessary to confirm our findings and to clarify the association between persistent oral HCMV infection and periodontitis progression.
